# An Examination of John Henryism in Adults Living with Sickle Cell Disease

**DOI:** 10.1007/s40615-024-02054-5

**Published:** 2024-07-08

**Authors:** Khadijah E. Abdallah, Kayla E. Cooper, Ashley J. Buscetta, Hasmin C. Ramirez, Harold W. Neighbors, Vence L. Bonham

**Affiliations:** 1 Social and Behavioral Research Branch, National Human Genome Research Institute, National Institutes of Health, 31 Center Drive, Suite B1B37, Bethesda, MD 20892, USA; 2 Rollins School of Public Health, Emory University, Atlanta, GA, USA; 3 Medical College of Georgia, Augusta, GA, USA; 4 School of Public Health, The University of Michigan, Ann Arbor, MI, USA; 5 School of Public Health and Tropical Medicine, Tulane University, New Orleans, LA, USA

**Keywords:** Sickle cell disease, John Henryism, Active coping, Sleep health, Biopsychosocial model, Chronic disease

## Abstract

**Background:**

John Henryism (JH) is a behavioral predisposition for high-effort coping with adversity. JH has been associated with hypertension in Black Americans with low socioeconomic status (SES) and is also found to be associated with psychological well-being. Sickle cell disease (SCD), a rare genetic disease largely affecting Black Americans in the United States, presents as a chronic condition that may benefit from a deeper understanding of the impact of JH on overall health.

**Purpose:**

This study examined the association between high and low JH and diastolic blood pressure, systolic blood pressure, hypertension prevalence, and sleep function. We relied on the biopsychosocial transaction model to adjust for relevant clinical and sociodemographic variables.

**Methods:**

This was a cross-sectional secondary analysis of 274 adults with SCD living in the United States and recruited between 2014 and 2020. Study visits consisted of physical examinations, medical history, demographic, and psychosocial questionnaires. Adjusted linear regressions estimated associations between high and low JH and diastolic and systolic blood pressure as well as self-reported sleep function. Multivariable logistic regression was used to examine associations with hypertension prevalence.

**Results:**

High JH was significantly associated with lower diastolic blood pressure (*β* = − 2.98; 95% confidence interval = − 5.92, − 0.04) but higher sleep dysfunction (*β* = 2.76; 95% confidence interval = 1.45, 4.07).

**Conclusions:**

Overall, we found positive psychological coping resources associated with high JH, with the exception of sleep.

**Trial Registration.:**

ClinicalTrials.gov Identifier: NCT02156102.

## Introduction

I am one of those people that when you tell me I can’t do something…I’m going to prove you wrong…that’s my coping (Study Participant)

The origins of John Henryism (JH) stem from the folktale of John Henry, a Black man who worked on railroads during the post-civil war era. After competing and winning against a steam drill in laying railroad track, John Henry died due to extreme mental and physical exhaustion [1]. At its core, JH metaphorically explicates the response of Black individuals to chronic environmental stressors such as systemic racism and racial prejudice [2–5]. John Henryism is, by definition, an *active, high-effort* strategy for coping with race-based socioeconomic adversity which has subsequent effects on cardiovascular health [6]. JH also interacts in complex ways with race, personal aspirations for employment success, gender, and sociocultural constructs [6].

Thankfully, the story of John Henry is just a fable, however, a prophetic story, nonetheless. The research by Dr. Sherman James, the originator of the theory and measure (John Henryism Active Coping Scale), has created a body of rigorous scientific evidence across disciplines that substantiates core biological truths from within this story [5–9]. The original work by James indicated that JH was related to blood pressure among Black Americans with low socioeconomic status under specific psychosocial conditions [5]. Further JH research by James’ and others revealed complex, within-gender, higher-order interactions having to do with the complicated self-reported interplay among success on the job, psychological investment in work, and the degree to which Black men and women felt that their race helped or hindered their job performance [6–9].

We identified limited published research that explored the impact of JH on chronic diseases and none in sickle cell disease (SCD) [2, 10]. SCD is a blood disorder caused by a single-point mutation in the beta-globin gene [11–14]. In the United States, SCD is considered a rare disease affecting approximately 100,000 individuals, primarily those of African descent with one out of 365 Black newborns diagnosed with the condition [15, 16]. There are multiple genotypes for SCD that range in disease severity [12–14]. A patient’s disease morbidity is a summation of both genetic and non-genetic modifiers (i.e., environmental factors, perceived stress, self-esteem, sleep hygiene, substance use, etc.) [11]. Individuals with SCD face a myriad of chronic cardiopulmonary, cerebrovascular, and musculoskeletal complications throughout their lifetime and can vary widely from patient to patient [13, 14]. Cardiopulmonary complications are some of the most common causes of mortality for patients with SCD, and sleep health is a known risk factor for cardiovascular diseases [17].

Managing physical and mental stress is paramount to reducing clinical and psychosocial complications associated with SCD [18]. As demonstrated by previous studies, psychological stress among individuals living with SCD is associated with higher vaso-occlusive pain episodes [19]. Moreover, individuals living with SCD report a relationship between increased pain episodes and higher levels of psychological issues including anxiety, depression, and emotional distress [20]. As such, it is understood that coping strategies can be employed as mechanisms of support for SCD patients combatting continuous clinical and psychological complications due to their disease [21]. Previous literature has highlighted the therapeutic capability of coping methods, such as journaling, diverting attention, and positive self-talk, which may improve mood and help prevent pain episodes, hospital admissions, and clinic or emergency department visits for patients with SCD [22]. Implementation of coping strategies has also shown improved psychological adjustment for SCD individuals, promoting cognitive restructuring, better pain management, and less negative thinking [23]. However, there are no studies exploring the relationship between the coping mechanism of JH and physical and psychological health among adults living with SCD.

The introductory quote from a study participant living with SCD describes their internal behavior of exerting maximum effort to cope with life obstacles. This is a classic characteristic associated with JH. Given prior research, there is reason to believe that high JH, *potentially* an adaptive coping response to stress, could be beneficial as a personal mechanism for managing chronic diseases such as SCD. The research suggests that the answer depends on socioeconomic position, gender, age, and marital status.

This study investigates sleep in this SCD population. There is a current understanding that individuals with SCD experience sleep disorders and sleep disturbances. Recently, there is limited emerging evidence on how sleep dysfunction impacts SCD. Sleep disturbance has been associated with poor cognition, depression, and worse quality of life [24–26]. Given that this is the first study of JH and sleep health in SCD, it is necessary to conduct an *exploratory* analysis of the measure and the research question that we seek to answer. We hypothesize that for adults living with SCD, the level of JH is highly relevant to health indicators, such as sleep quality, because JH reveals the “degree to which an individual is predisposed to take on difficult stressors with determined, high-effort coping that is also optimistic” [27]. Therefore, we contend that the JH findings most relevant to SCD have to do with personal investment, race, and expectations/hope of successful chronic disease self-management.

## Methods

### Setting and Study Design

Our study consisted of 274 participants and utilized psychosocial and clinical data from the INSIGHTS “Insights into the Microbiome and Environmental Contributions to Sickle Cell Disease and Leg Ulcers Study” (Clinicaltrials.gov: NCT02156102). The INSIGHTS Study is a cross-sectional study that broadly analyzes the microbial, genomic, psychosocial, clinical, and environmental factors that influence the phenotypic variation of adults living with SCD. Participants were recruited for the INSIGHTS Study through SCD-related events and conferences, hematologist referrals, social media advertising, and snowball sampling. Participants were enrolled between June 2014 and January 2020, at either the National Institutes of Health’s Clinical Center in Bethesda, Maryland, or Montefiore Medical Center in the Bronx, New York.

### Participants and Selection Criteria

All participants in the INSIGHTS Study were adults (18 years or older) with a clinical diagnosis of SCD. Each participant provided a complete medical history and underwent a comprehensive physical exam. Participants were also administered a variety of psychosocial measures and demographic questionnaires (sex assigned at birth, race/ethnicity, marital status, current employment status, education, and age).

### Predictors and Outcome

**Fig. 1:**
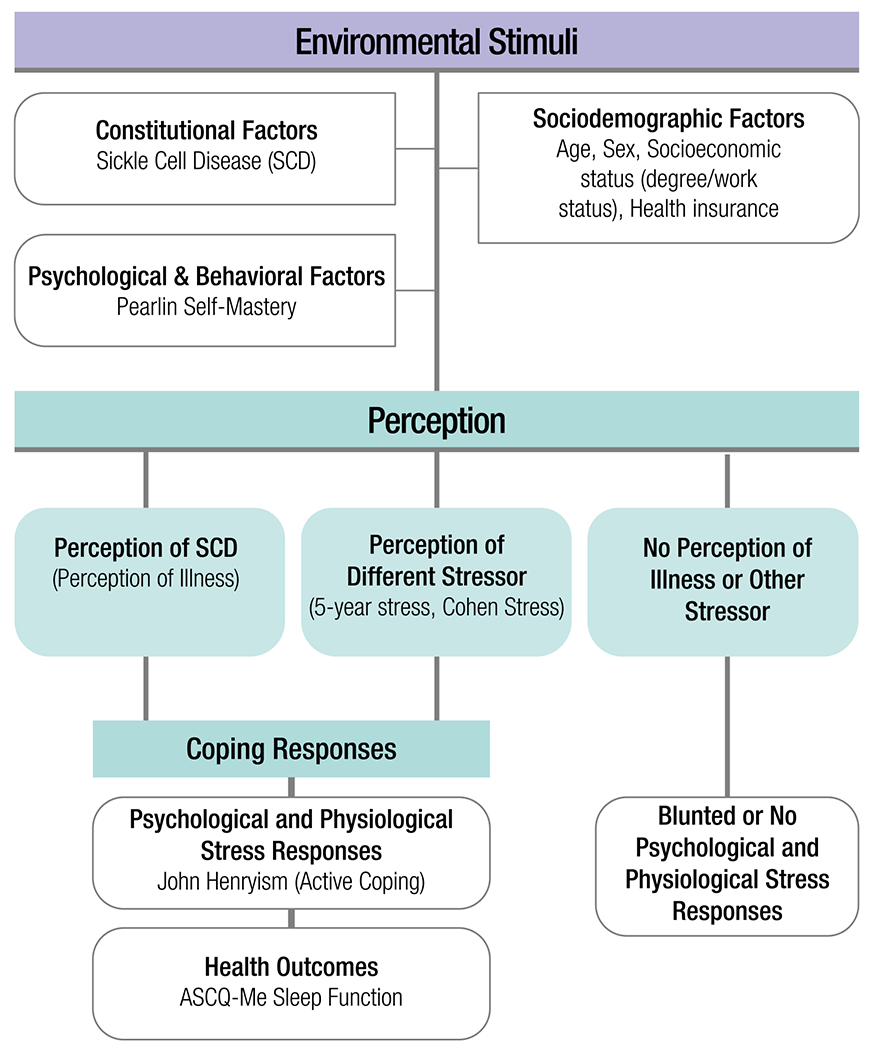
A conceptual model to examine the biopsychosocial effects of perceived illness and coping strategies on sleep

This study was designed to evaluate and quantify the relationship between sociodemographic, psychological, and behavioral factors associated with the perception of SCD-based stressors and other stressors on health outcomes. A modified version of the biopsychosocial transaction model was used to conceptualize the predictors for this study [28] (Fig. 1).

#### Outcome Measure

Sleep quality was the main health outcome that we examined for this study (Fig. 1). Sleep quality was determined through the ASCQ-Me Sleep Measure which identifies participant sleep quality based on scoring from the ASCQ-Me scale development SCD reference cohort [29]. The sleep quality score was determined from participant responses to the five-item sleep measure, and questions measured sleep patterns within the past 7 days. Scores range from 5 to 25, and higher scores indicate more sleep dysfunction (i.e. poor sleep quality) [29].

#### Predictors

The *John Henryism Active Coping Scale* (JHAC12) was the primary predictor of interest in this study. It was used to measure the determination to succeed in response to chronic stressors among participants [5, 6]. JHAC12 scores were measured from participant responses to a 12-item Likert scale. Questions measured participants’ coping styles when faced with difficult psychosocial and environmental stressors. Items on the scale include “Once I make up my mind to do something, I stay with it until the job is completely done,” “I’ve always felt that I could make of my life pretty much what I wanted to make of it,” and “Hard work has really helped me get ahead in life.” Scores range from 12 to 60. Low and high JHAC12 categorization was based on the median JHAC12 score of 52 for this study sample. Scores between 12 and 52 were considered low JHAC12 utilization, and scores slightly higher than 52, between 52.09 and 60, were considered high JHAC12 utilization. This dichotomization was modeled on Dr. Sherman James’ pioneering studies of JH [9]. The JHAC12 scale has an internal consistency (Cronbach’s alpha) of 0.76 for this study sample.

Other psychosocial predictors include *locus of control*, which was evaluated using the Pearlin Self-Mastery Scale [30]. This scale measures the extent to which individuals perceive themselves as in control of the happenings in their lives, both currently and in the future. *Perception of Illness* was captured through the Brief Illness Perception measure [31]. This measure is utilized in chronic disease populations and was modified for individuals living with SCD for the present study. Questions measure the perceived impact of SCD on participants’ lifestyle.

The *Acute Stress* measure was a count of stressful life-related events that had occurred over the past 5 years (e.g., death of a relative, financial problems, assaults/burglary) [32]. *Psychological stress* was measured through the Cohen Global Perceived Stress Scale [33].

Replicating the original JH studies, we included SES as a variable. For our study purposes, the SES indicators examined were employment status and education level. Individuals with a bachelor’s degree or higher and employed were considered high SES.

### Statistical Analysis

We used descriptive statistics to examine the sociodemographic and psychosocial factors in our population. We also used descriptive statistics to examine frequencies across the low and high JH groups in the population. Bivariate analyses utilizing chi-squared and nonparametric *T*-tests were used to determine whether certain characteristics of the study participants were associated with the JH categorization. For the adjusted models, multivariable linear regressions were used to examine the association between the predictors and sleep quality/dysfunction. Regression diagnostics were performed, residual distributions were examined, and no concerning values were detected. Missing data were addressed through listwise deletion and assessed for bias; a total of 217 participants had complete data. Statistical significance for all results was determined based on a *p*-value of < 0.05, and 95% confidence intervals are reported. All analyses were performed using SAS version 9.4.

## Results

### Demographics

Demographic and clinical characteristics’ data can be found in Tables 1 and 2, respectively. The sample consisted of 274 adults with SCD aged 19–71 with a mean age of 39 years (SD ± 12.1). The cohort was predominantly African American (97%), biologically female (56%), insured (91%), above the poverty line (76%), current nonsmokers (95%), and unmarried (68%). Other demographic data indicated that a substantial portion of this population was educated with a bachelor’s degree or higher (40%), currently employed (43%), and had a household income greater than $50,000 (33%). The JH categories were evenly divided for this cohort (50% high, 50% low). Table 2 presents the participants’ clinical data. The average reported pain score for the past 7 days was 3.8 (SD 2.8) on a scale of 0–10. The majority of the study population did not report current obstructive sleep apnea (87%), and the mean BMI was 25.3 (SD 6.0). Additionally, most participants have homozygous sickle cell anemia, HbSS (79%), one of the more severe SCD genotypes, followed by HbSC (13%), HbSB + (5%), and HbSB0 (3%).

### Bivariate Analyses

The bivariate analyses present comparisons across the demographic, clinical, and psychosocial characteristics between the low and high John Henryism categories. For the demographic characteristics (i.e., age, marital status, insurance status, poverty frequency), there were no significant differences between the low and high JH cohorts (Table 3).

#### Clinical Measures

There were no significant differences in the bivariate analyses across the John Henryism categories for the clinical measures of BMI and sleep function (*p* > 0.05). The results also indicated no significant difference across the low and high JH categories with respect to current obstructive sleep apnea (*p* > 0.05).

#### Psychosocial Measures

There was a positive association between high JH and lower self-reported stress; high JH was more likely for individuals with lower mean scores on the global measure of Perceived Stress Scale (PSS) (*p* = 0.0001). High JH was also more likely for individuals with higher mean Pearlin self-mastery scores (*p* = 0.01). Marginal significance was noted for the 5-year stress index (*p* = 0.06) and illness perception scores (*p* = 0.07).

### Multivariable Regression Model

John Henryism was the predictor of interest for the sleep model. An important finding is that study participants in the high JH group exhibited higher average sleep dysfunction scores than those in the low JH group by 2.76 points (95% confidence interval = 1.45, 4.07; *p* ≤ 0.001) (Table 4). There was variability in the association of the sociodemographic, psychosocial, and clinical factors with the sleep dysfunction measure. The demographic variables that were significantly noted with the sleep outcome were age and SES. High SES was significantly associated with lower mean sleep dysfunction scores (*β* = − 2.13; 95% confidence interval = − 3.46, − 0.80; *p* < 0.01), while age was associated with higher sleep dysfunction scores (*β* = 0.07; 95% confidence interval = 0.01, 0.13; *p* < 0.05). The sleep dysfunction measure was also associated with the psychosocial measures. Higher brief illness perception (*β* = 0.11; 95% confidence interval = 0.04, 0.18; *p* ≤ 0.01), higher perceived stress (*β* = 0.24; 95% confidence interval = 0.10, 0.37; *p* ≤ 0.01), and higher reported 5-year stress index (*β* = 0.47; 95% confidence interval = 0.17, 0.76; *p* ≤ 0.01) were all significantly associated with higher sleep dysfunction scores. The sleep quality model accounted for 37% of the variation in the outcome.

## Discussion

While the majority of previous literature on JH focuses on cardiovascular disease, this is the first study, to our knowledge, that examines the association between active coping mechanisms and sleep health in SCD.

The foundational studies of JH established its association with blood pressure among African Americans living in the coastal plains of North Carolina [5, 7]. John Henryism has shown a complicated, multidirectional relationship to cardiovascular diseases [6]. On one hand, the story can be read as overcoming systemic racism, or as a victory over automation. These “victories” seem to *suggest* that working as hard as possible against all odds is good for quality of life. On the other hand, JH can contribute to hypertension (HTN) and as a result, premature death. Given that 97% of respondents in the present study identified as Black or African American, interpreting these findings in light of the initial studies on JH is relevant to this, as well as future research. Our study drew on existing literature to define high and low JH using the median score of 52 for this study sample, which is comparable to other works on JH [6, 8, 34].

Our analysis was guided by the biopsychosocial transaction model exploring how active coping may be beneficial to individuals with SCD as they navigate the burden of their chronic condition along with other life stressors. However, its potential benefit was not found for the sleep outcome of this study. Ultimately, we found that JH was positively associated with the sleep measure; individuals with high JH self-reported higher average scores in poor sleep quality than individuals with low JH. More specifically, individuals with high JH (or, are actively coping with their circumstances) report more difficulty falling asleep, staying asleep, and getting enough sleep, indicating a potential tendency for JH to act as a trigger for sleep dysfunction. Among individuals living with SCD, general studies on SCD and sleep for adults have been few and varied in their assessments of sleep but largely point to the existence of sleep dysfunction [24, 35]. Few studies, though, have measured psychosocial predictors of sleep health aside from Moscou-Jackson et al., where researchers examined, among other factors, the impact of stress on insomnia in individuals with SCD but reported no statistical significance between varying levels of stress and sleep hygiene [35]. Compared to that study, our study found some preliminary association between Cohen’s Global Perceived Stress and the 5-year stress index with increased sleep dysfunction. Reasons for this may include the comprehensiveness of our stress measures and/or the different sleep scales employed by our study. The ASCQ-Me sleep measure used here is a tool that was developed and validated in a population of U.S. adults with SCD [29]. It is also noteworthy to state that the gold standard for measuring sleep quality includes actigraphic and/or polysomnographic assessments. To date, however, only one study comparing subjective sleep quality to the gold standards among adults with SCD was investigated by Sharma and colleagues, with findings indicating that 44% of adults with SCD who conducted overnight polysomnography following self-reported measures of sleep were found to have poor sleep quality [36]. Thus, while our study utilized self-reported scales, which is necessary to capture certain aspects of sleep health (e.g., subjective sleep quality), more studies are needed to conduct longitudinal and gold-standard assessments of sleep disorders among individuals with SCD.

Furthermore, although earlier studies on JH among Black Americans focused on blood pressure and other aspects of cardiovascular health, they lend further credibility to our findings that while high JH can at times, be beneficial, in other instances, it may worsen disease burden for patients with chronic diseases [37]. Additionally, outside of the context of SCD, no studies, to our knowledge, have examined the association between JH and sleep, but a few hypothesize JH’s potential role as a stressor in explaining their findings on sleep dysfunction [38, 39]. To that end, larger studies are needed, and future directions should continue to explore the relationship between JH and sleep across other chronic conditions to expand the literature on active coping. These findings can provide potential insight to inform psychosocial interventions to improve disease morbidity of adults with SCD.

## Limitations

The limitations of our study must also be considered. Causal inference is not possible given the cross-sectional design of this study. Future studies should examine these factors in a longitudinal manner. Additionally, the ASCQ-Me sleep quality is a self-reported measure, and sleep dysfunction was not clinically diagnosed; future assessments should also include methods that rely on clinical judgment to assess sleep dysfunction [40]. As this study is exploratory in nature and focused on psychosocial outcomes, we cannot address how a patient’s medications or other treatments/ therapies may alter JH scores and subsequent survey responses. Future studies should address how clinical data (i.e., treatments, laboratory studies) are impacted by high or low JH coping styles. Additionally, it would be remiss to negate the socioeconomic and sociopolitical climate that may influence a participant’s JH coping score during the period of data collection. However, the JH coping measure does account for how a participant copes with their circumstances broadly which is contextualized through a social, economic, and political lens. Future studies may also want to consider examining other SES indicators including income, occupation, childhood SES, cumulative SES, and neighborhood SES to strengthen the operationalization of this construct [41]. Despite the limitations, the strengths of our study include highlighting the importance of studying JH among individuals living with SCD and possibly across other disease populations. There is generally a dearth of research around SCD and health outcomes. For hypertension and sleep outcomes, most SCD research has focused on pediatric cases or the predictive role of sleep for pain outcomes among adults with SCD [24, 42, 43]. Considering that SCD is a rare disease, most studies are limited in sample size including this study.

## Conclusion

This study seeks to understand multifactorial mechanisms that affect different aspects of this population’s health and well-being. This is the first study to examine JH within a population living with SCD. Sickle cell disease was the first molecular genetic disease identified [44]. It is a chronic disease with a long-interconnected history with discrimination, health inequities, and race in the United States. Individuals living with sickle cell disease are required to establish approaches to cope with their disease. In summary, we found positive psychological coping resources associated with high JH, including personal control and lower self-reported stress. However, individuals in the high JH group exhibited higher average sleep dysfunction scores than those in the low JH group [45]. Further research is required to understand the role of JH active coping in the management of the mental and physical health of individuals living with sickle cell disease.

## Figures and Tables

**Table 1:** Demographic characteristics *(n* = *274)*

	n^a^	%	SD^b^	Range

Race/ethnicity				
Non-African American	8	3	–-	–-
African American	256	97	–-	–-
Hispanic and/or Latino	28	10	–-	–-
Health insurance coverage				
Yes	247	91	–-	–-
No	24	9	–-	–-
Marital status				
Unmarried	183	68	–-	–-
Married	86	32	–-	–-
Gender				
Male	120	44	–-	–-
Female	154	56	–-	–-
Education				
< Highschool and highschool	55	20	–-	–-
Some college	106	39	–-	–-
Bachelors or >	109	40	–-	–-
Living in poverty				
No	179	76	–-	–-
Yes	56	24	–-	–-
Household income ≥ 50 K	84	33	–-	–-
Currently employed	117	43	–-	–-
Current smoker	14	5	–-	–-
John Henryism				
Low John Henryism	136	50	–-	–-
High John Henryism	134	50	–-	–-
	n	M^c^	SD	Range
Age	273	38.8	12.1	19–71
John Henryism	270	51.7	5.4	31–60
ASCQ-Me sleep quality	273	13.4	5.8	5–25
Beck depression	261	10.9	8.9	0–49
Global Cohen’s perceived stress	259	19.8	7.0	5–41
5-year stress index, mean (SD)	254	4.3	2.3	0–10
Brief illness perception	267	45.0	11.4	7–70
Pearlin self-mastery	266	25.5	5.6	10–35

a *n* number of participants

b *SD* standard deviation

c *M* mean

**Table 2:** Clinical Characteristics *(n=274)*

	n^a^	%	SD^b^	Range
**Genotype**				
HbSS	217	79	---	---
HbSC	35	13	---	---
HbSB+	13	5	---	---
HbSB0	8	3	---	---
**Obstructive Sleep Apnea**				
No	233	87	---	---
Yes	32	12	---	---
	n	M^c^	SD	Range
**Diastolic BP**	273	67.9	10.7	42 – 117
**Beck Depression**	261	10.9	8.9	0 – 49
**Pain Score (past 7 days)**	275	3.8	2.8	0 – 10
**BMI**	267	25.3	6.0	12.4 – 50.0

a *n* number of participants

b *SD* standard deviation

c *M* mean

**Table 3:** Characteristics of the study population comparing high and low John Henryism

	*n = 136*	Low	*n = 134*	High	*p*-value
Age-mean (SD)	135	38.8 (12.1)	132	38.9 (12.4)	.99
Gender (%)					.54
Male	62	46%	56	42%	
Female	73	54%	77	58%	
Race/ethnicity (%)					.72
African-American	127	96%	125	98%	
Non-African American	5	4%	3	2%	
Highest education (%)					.97
High school and/or below	27	20%	26	20%	
Some college	51	38%	53	40%	
Bachelor’s degree and above	55	41%	54	41%	
Poverty status (%)					.44
Yes	24	21%	30	26%	
No	91	79%	86	74%	
Household income ≥ 50 K (%)	42	34%	42	34%	.75
Work status (%)					.14
Currently employed	52	39%	64	48%	
Not currently employed	81	61%	68	52%	
Marital status (%)					.79
Married	44	33%	41	31%	
Not married	89	67%	91	69%	
Health insurance coverage (%)					.83
Yes	123	92%	121	91%	
No	11	8%	12	9%	
Current smoker (%)					.59
Yes	6	4%	8	6%	
No	130	96%	126	94%	
BMI, mean (SD)	132	25.1 (5.4)	129	25.5 (6.5)	.81
Diastolic blood pressure, mean (SD)	134	69.0 (10.6)	133	66.7 (10.6)	.09
ASCQ-Me sleep functionality, mean (SD)	135	12.9 (5.6)	133	13.9 (6.0)	.17
Obstructive sleep apnea (%)	15	11%	16	12%	.79
Brief illness perception, mean (SD)	132	46.2 (10.5)	130	43.6 (12.2)	.07
Global Cohen’s perceived stress, mean (SD)	131	21.5 (6.7)	126	17.9 (6.9)	.0001
5-year stress index, mean (SD)	126	4.5 (2.3)	123	4.0 (2.4)	.06
Pearlin self-mastery score, mean (SD)	133	24.6 (5.2)	128	26.3 (6.0)	.01

*SD*, standard deviation

**Table 4:** Associations between study characteristics and ASCQ-Me sleep function

	ASCQ-Me sleep functionality	
	Estimates (SE)	95% CI^a,b^
**John Henryism**	2.76 (0.7)	[1.45, 4.07]***
High vs. low		
**Gender**	0.16 (0.7)	[− 1.19, 1.51]
Male vs. female		
**Age**	0.07 (0.03)	[0.01, 0.13]*
**SES**	− 2.13 (0.7)	[− 3.46, − 0.80]**
High vs. low		
**Married**	− 0.07 (0.8)	[− 1.56, 1.42]
Yes vs. no		
**Health insurance coverage**	− 0.32 (1.1)	[− 2.50, 1.86]
Yes vs. no		
**Brief illness perception**	0.11 (0.03)	[0.04, 0.18]**
**Cohen perceived stress**	0.24 (0.1)	[0.10, 0.37]**
**5-year stress index**	0.47 (0.1)	[0.17, 0.76]**
**ASCQ-Me sleep functionality**	–-	–-
**Pearlin self-mastery score**	0.01 (0.1)	[− 0.15, 0.17]
**BMI, kg/cm^2^**	− 0.04 (0.1)	[− 0.16, 0.07]
**Diastolic blood pressure**	0.06 (0.03)	[− 0.01, 0.12]^¥^
*F*-statistic for the model	9.66***	–-
*R*-square for the model	0.37	–-

a The parameter estimate gives the change in the continuous outcome measures (blood pressures and/or sleep functionality) for every unit increase (e.g., every year increase in age) or between the indicated group and reference group (e.g., high John Henryism vs. low)

b The parameter estimate gives the probability of having hypertension for every unit increase (e.g., every year increase in age) or between the indicated group and the reference group (e.g., high John Henryism vs. low)

¥ *p* < 0.10,

* *p* < 0.05,

** *p* ≤ 0.01,

*** *p* ≤ 0.001

## Data Availability

Data will be made available upon request.
